# Clinicopathological Analysis of Complicated Colorectal Cancer: A Five-Year Retrospective Study from a Single Surgery Unit

**DOI:** 10.3390/diagnostics13122016

**Published:** 2023-06-09

**Authors:** Elena Savu, Liviu Vasile, Mircea-Sebastian Serbanescu, Dragos Ovidiu Alexandru, Ioana Andreea Gheonea, Daniel Pirici, Stefan Paitici, Stelian Stefanita Mogoanta

**Affiliations:** 1 Doctoral School, University of Medicine and Pharmacy of Craiova, 200349 Craiova, Romania; 2 Department of Oncopediatrics, Clinical Emergency County Hospital, 200642 Craiova, Romania; 3 Department of Surgical Semiology, Faculty of Medicine, University of Medicine and Pharmacy of Craiova, 200349 Craiova, Romania; vliviu777@yahoo.com; 4 Third General Surgery Department, Clinical Emergency County Hospital, 200642 Craiova, Romania; stefanppaitici@gmail.com (S.P.); ssmogo@yahoo.com (S.S.M.); 5 Department of Medical Informatics, Faculty of Medicine, University of Medicine and Pharmacy of Craiova, 200349 Craiova, Romania; 6 Department of Biostatistics, Faculty of Medicine, University of Medicine and Pharmacy of Craiova, 200349 Craiova, Romania; 7 Department of Radiology and Medical Imaging, Faculty of Medicine, University of Medicine and Pharmacy of Craiova, 200349 Craiova, Romania; iagheonea@gmail.com; 8 Department of Histology, Faculty of Medicine, University of Medicine and Pharmacy of Craiova, 200349 Craiova, Romania; daniel.pirici@umfcv.ro; 9 Department of General Surgery, Faculty of Dental Medicine, University of Medicine and Pharmacy of Craiova, 200349 Craiova, Romania

**Keywords:** colorectal cancer, colorectal cancer complications, adenocarcinoma, histopathology, lymph nodes, vascular invasion, perineural invasion

## Abstract

Patients with primary colorectal cancer can present with obstructions, tumor bleeding, or perforations, which represent acute complications. This paper aimed to analyze and compare the clinical and pathological profiles of two patient groups: one with colorectal cancer and a related complication and another without any specific complication. We performed a five-year retrospective study on colorectal cancer patients admitted to a surgery unit and comparatively explored the main clinical and pathological features of the tumors belonging to the two groups. A total of 250 patients with colorectal cancer were included in the analysis. Of these, 117 (46.8%) had presented a type of complication. The comparative analysis that examined several clinical and pathological parameters showed a statistically significant difference for unfavorable prognosis factors in the group with complications. This was evident for features such as vascular and perineural invasion, lymph node involvement, pathological primary tumor stage, and TNM stage. Colorectal cancers with a related complication belonged to a group of tumors with a more aggressive histopathologic profile and more advanced stages. Furthermore, the comparable incidence of cases in the two groups of patients warrants further efforts to be made in terms of early detection and prognosis prediction of colorectal cancer.

## 1. Introduction

Colorectal cancer (CRC), which includes colon and/or rectum cancer, poses a significant health concern in terms of morbidity and mortality. Globally, it is the third most frequently diagnosed cancer and the second leading cause of cancer-related deaths. [1].

According to the Globocan data for colorectal cancer burden in 2020, it was estimated that there were more than 1.9 million new cases and 0.9 million deaths worldwide, and it is expected that the global incidence of new colorectal cancer cases will reach 3.2 million by 2040 [2]. Regarding the European cancer burden, colorectal cancer was estimated to be the second most diagnosed cancer in Europe considering both sexes together.

The estimated data for Romania, extracted from national cancer registries, are superimposed with the European colorectal cancer pattern, with CRC representing the third localization as incidence in men and the second as mortality in this category, while in women it is ranked as the second localization in both incidence and mortality [3].

There are several recognized risk factors associated with the development of colorectal cancer, including advancing age, male sex, family history of colorectal cancer, inflammatory bowel disease, or modifiable lifestyle and nutritional factors; growing evidence also implicates the gut microbiome in colorectal cancer development and progression [4]. Moreover, the increasing incidence of early-onset colorectal cancer (before the age of 50 years) is an emerging trend [5].

Colorectal carcinogenesis is characterized by a gradual progression from adenoma to invasive carcinoma, and this timeline offers the opportunity to detect and remove colorectal adenomas via screening, thus preventing the progression to invasive carcinoma [1]. Individuals with a history of adenoma, colon cancer, or inflammatory bowel disease or those with a significant family history of CRC or adenoma, as well as those with an inherited cancer syndrome, are considered to be at high risk for colon cancer and should undergo active screening [6]. Both European and American evidence-based cancer guidelines recommend screening for colorectal cancer in all adults aged 50 to 74 years; furthermore, the United States Preventive Services Task Force advocates for lowering the starting age for screening from 50 to 45 years [6,7]. There are a multitude of options for CRC screening. Stool-based tests include the guaiac-based fecal occult blood test (gFOBT), the fecal immunochemical test (FIT) and multitargeted stool DNA testing (FIT-DNA). Invasive techniques include flexible sigmoidoscopy or colonoscopy screening. While the application of these strategies is not uniform across European countries, in the United States, all the available screening methods are being offered [7,8].

Among the prognostic factors in colorectal cancer, the AJCC/UICC tumor node metastasis (TNM) stage remains the gold standard of prognostic factors in CRC. Other important morphological prognostic factors refer to lymph node status, tumor grade, and assessment of lymphatic, venous, and perineural invasion or significant (grade of >1) tumor budding [6,9]. Several parameters are associated with a higher risk for recurrence or distant metastasis development: lymph node sampling of less than 12, pT4 stage (including tumor perforation), or tumor presentation with obstruction [6].

The therapeutic approach in colorectal cancer is complex, and it is ideally guided by a multidisciplinary team that includes a general surgeon, medical oncologist, radiation oncologist, pathologist, and radiologist. Currently, patients with advanced CRC are mainly treated by surgery combined with neoadjuvant chemotherapy, adjuvant chemotherapy, and radiotherapy to improve surgical treatment and also by adding various targeted therapies guided by the tumor molecular profile [1,10].

A significant proportion of patients with colon and rectal cancer still present in advanced and complicated stages, with the most common causes being obstruction (78%), perforation (10%), and inferior digestive hemorrhage (4%) [11].

In this study, we aimed to analyze the clinicopathological characteristics of a population of patients with colorectal cancer admitted to a surgery department within a tertiary care hospital over five years, between January 2018 and December 2022, and that presented with one of the main complications that may occur in the colorectal cancer setting, i.e., large bowel obstruction, perforation, or hemorrhage.

## 2. Materials and Methods

We conducted a comparative retrospective study that included patients diagnosed with complicated and uncomplicated colorectal cancers admitted to the Third Department of General Surgery, Clinical Emergency County Hospital Craiova.

Patients were enrolled in the current retrospective study if their hospital admission in the surgery unit occurred between January 2018 and December 2022. For data collection, we searched the clinical observation sheets and digital records belonging to the hospital’s electronic database.

The demographic information, type of hospital admission, type of complication, surgeries performed, and other clinical data were identified from digital and paper records.

Several inclusion criteria were considered for the study participants: the patients were aged 18 years or older; they had undergone a type of surgical intervention, either resection or palliative surgery; they had a confirmed diagnosis of colon or rectal cancer, supported by a histopathology report (that provided information about the type of tumor, grading, and classification in the pTNM system); the diagnoses were based on the International Classification of Diseases (ICD-10) coding system. Patients admitted for evaluation or patients admitted for bowel transit reconstruction after a previously temporarily performed stoma were excluded. Other exclusion criteria were patients with benign colorectal pathology or patients for whom no pathological reports were found.

The Ethical Committee of the Clinical Emergency County Hospital Craiova was informed and this study was approved (approval number 60914 from 30 December 2022) on the following bases: (1) data were collected as part of a retrospective, observational study; (2) the study did not interfere with current medical care; (3) no experimental substances were administered to the patients and no biological samples were collected as part of the study; and (4) data were collected and analyzed in an anonymized manner so that patients’ data confidentiality was not breached.

The following variables were collected: (1) background characteristics (age, gender, and place of origin); (2) colorectal cancer characteristics (tumor location, type of complication, pathological features of the resected tumor, and presence or absence of distant metastases); and (3) presence of associated comorbidities in the analyzed patients.

The pathological features assessed included tumor dimension, histopathological type and subtype, tumor differentiation grade, tumor stage, vessel and perineural invasion, and lymph node status. The tumor differentiation grades were classified as well-differentiated, moderately differentiated, and poorly differentiated. The tumor stage was determined according to the eighth edition of the “TNM Classification of Malignant Tumours” from the Union for International Cancer Control [12].

For comparison and further analysis, patients were divided into two groups according to presence or absence of a complication associated with colorectal cancer. The complications referred to intestinal obstruction, perforation, and bleeding.

For statistical analysis, we used Microsoft Excel 2019 MSO (version 2304 Build 16.0.16327.20200), to create a comprehensive database in which we included all the variables of interest, and we used MedCalc statistical software version 20.218 to perform the statistical evaluation. Frequencies were presented as absolute numbers of cases and percentages. Chi-squared tests were used to compare ordinal or nominal variables. Continuous variables were compared using the Mann–Whitney *U*-test if the variable was not distributed normally. A *p*-value of less than 0.05 was considered statistically significant.

## 3. Results

**Figure 1:**
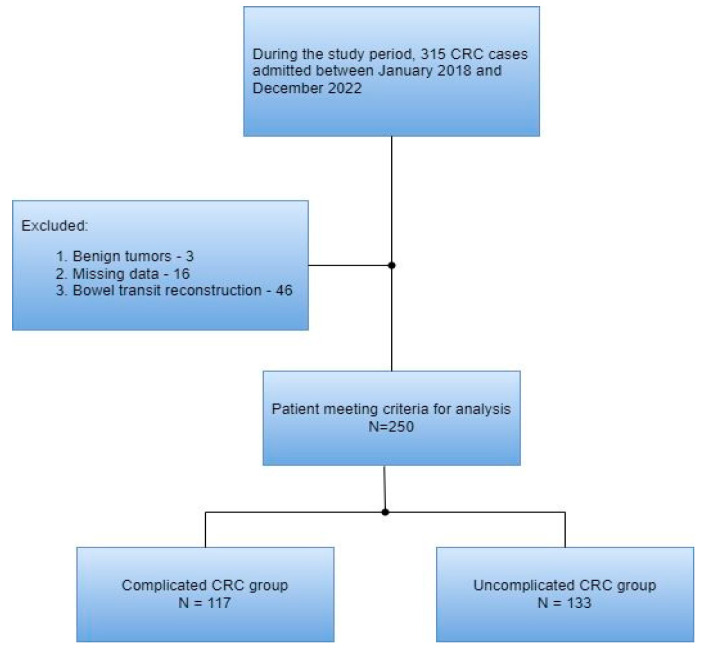
Flow diagram of the patient selection process.

A total of 315 patients with colorectal cancer were included in this study’s cohort. Of these, 65 were excluded according to the exclusion criteria. Thus, 250 patients were included in the final analysis (Figure 1).

**Figure 2:**
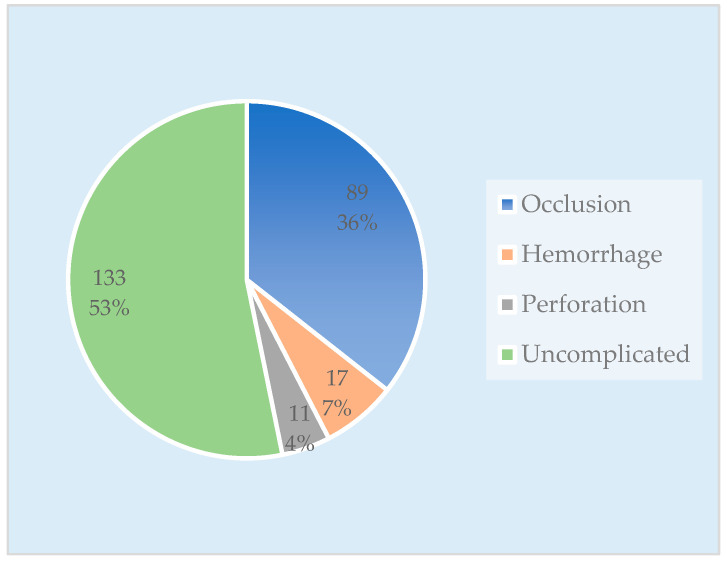
Chart with the number of cases in each patient group.

The group with complications consisted of 117 patients, categorized as follows: 89 cases (36%) presented with occlusions, 17 cases (7%) presented with hemorrhages, and 11 cases (4%) presented with tumor perforations. The comparison (uncomplicated) CRC group included 133 patients (Figure 2).

A comparison of the characteristics of patients with a colorectal cancer-related complication against those of patients without complications is shown in Table 1. A group difference analysis was performed to evaluate which parameters differed between the patients in the complicated CRC group and the patients in the uncomplicated CRC group.

There was no statistical difference in the sex ratio between the two groups (*p* = 0.232). The male-to-female ratio was 67:50 in the complicated CRC group. The number of male patients in both groups was greater than that of women. The mean age in both groups was very similar, approximately 67 years, with the same age range for both groups (between 41 and 86 years old).

No statistical significance was found regarding the place of origin in the comparison groups (*p* = 0.214), though a higher proportion of patients presenting a complication originated from rural areas (53% vs. 45.1%).

The initial tumor locations were represented by nine sites (cecum, ascending colon, hepatic flexure, transverse colon, splenic flexure, descending colon, sigmoid colon, rectosigmoid junction, and rectum); to simplify the final analysis, a more concise classification was elaborated, resulting in four categories: right colon, transverse, left colon, and rectosigmoid junction and rectum (*p* = 0.184). In both groups, the predominant locations of the tumors were attributed to the rectosigmoid junction and rectum, followed by the left colon tumors.

Regarding the type of surgery, the difference between the two groups was statistically significant (*p* = 0.0003), with a slightly higher proportion of radical surgery procedures performed in the uncomplicated group (74.4% vs. 72.6%), whereas palliative procedures were more prevalent in the group with complications (23.9% vs. 10.5%). The mean tumor size was similar between the two groups (4.7 vs. 4.78, *p* = 0.145).

**Figure 3:**
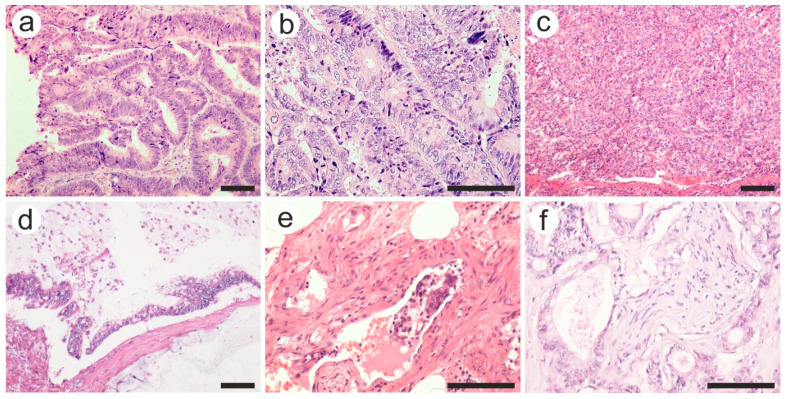
Various pathological features of colon carcinoma: (**a**) well, (**b**) moderate, and (**c**) poorly differentiated adenocarcinoma; (**d**) mucinous carcinoma with extracellular mucin accumulation; (**e**) intravascular tumor embolus in a venule, and (**f**) perineural invasion. Hematoxylin-eosin staining was used. The scale bars represent 50 µm. The images were used with the permission of, and were provided courtesy of, S.S.M and N.D.P., respectively.

Data from the histological reports revealed a predominance of adenocarcinomas (98.5% of cases), two neuroendocrine tumors, and only one leiomyosarcoma. The vast majority of histological subtypes were represented by adenocarcinoma NOS in both groups (Figure 3a–c), followed by mucinous adenocarcinomas (Figure 3d) and adenoma-like adenocarcinoma. No statistically significant difference was found when comparing the histopathological subtypes in both groups (*p* = 0.632). Regarding tumor grading, the most prevalent were medium differentiated tumors (grade 2, G2) for both groups (*p* = 0.252).

As for the pT category, more advanced tumors (T3 and T4 tumors) were found in the complication group, and the difference was statistically significant (*p* = 0.002). Along with the extension through the layers of the colon wall, tumor aggressiveness was translated as intravascular tumor emboli or perineural invasion (Figure 3e,f). Tumors with vascular and perineural invasion were more frequent among the complication group (27.7% vs. 12.5% and 32.5% vs. 13.5%, respectively), with a statistically significant difference (*p* = 0.010 and 0.002, respectively).

**Figure 4:**
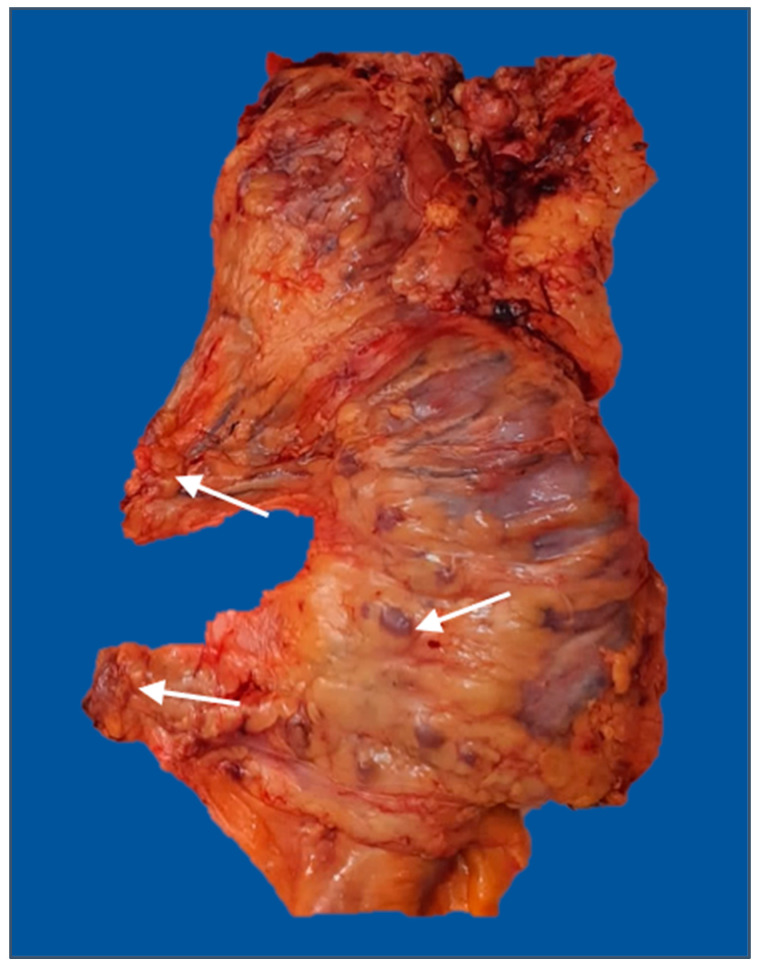
Right hemicolectomy specimen (posterior view) with enlarged lymph nodes in the mesocolon, middle colic, and right colic pedicles (arrows). The image was used with the permission of, and was provided by, S.S.M.

The mean number of retrieved lymph nodes in the subgroup with more than 12 harvested lymph nodes was 17.59. There was a greater proportion of cases with more than 12 retrieved lymph nodes in the uncomplicated subgroup (50.5% vs. 35.4%, *p* = 0.043). Furthermore, the complication group had a greater prevalence of invaded lymph nodes than the comparative group (56.1% vs. 38.7%, *p* = 0.021). Multiple pathological lymph nodes in pericolic soft tissue are indicated in Figure 4.

No difference was found when comparing the N and M categories among the two groups, but the proportion of cases in the complication group with advanced (y)pTNM stages (III and IV) was significantly higher than that in the uncomplicated group (*p* = 0.001).

No statistically significant difference was found when analyzing the number of cases during the five-year study period (*p* = 0.243), although the total number of colorectal cancer patients admitted to the unit increased in 2022 (65 identified cases). Several comorbidities were analyzed, and statistically significant differences were observed for diabetes mellitus, obesity, and the presence of another neoplasm. Obesity was more prevalent among patients with complications (*p* = 0.017).

The number of cases distributed by type of complication in relation to clinical and pathological features is detailed in Table 2.

**Figure 5:**
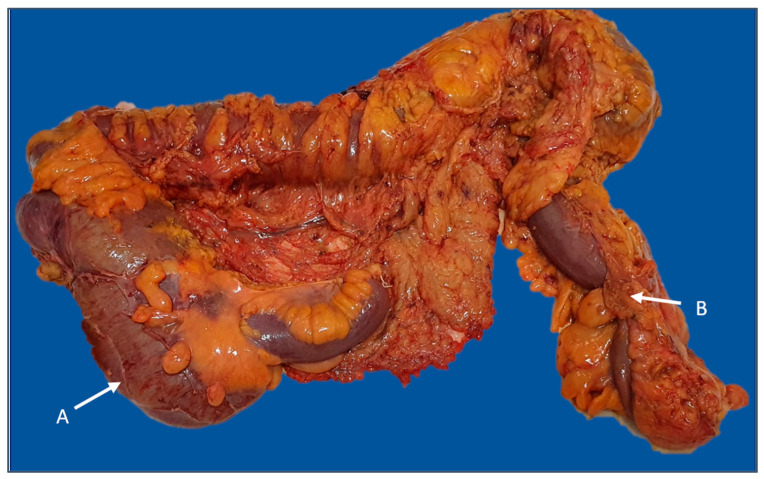
Subtotal colectomy specimen with imminence of cecal diastatic perforation (A) secondary to sigmoid obstructive cancer (B). The image was used with the permission of, and was provided by, S.S.M.

**Figure 6:**
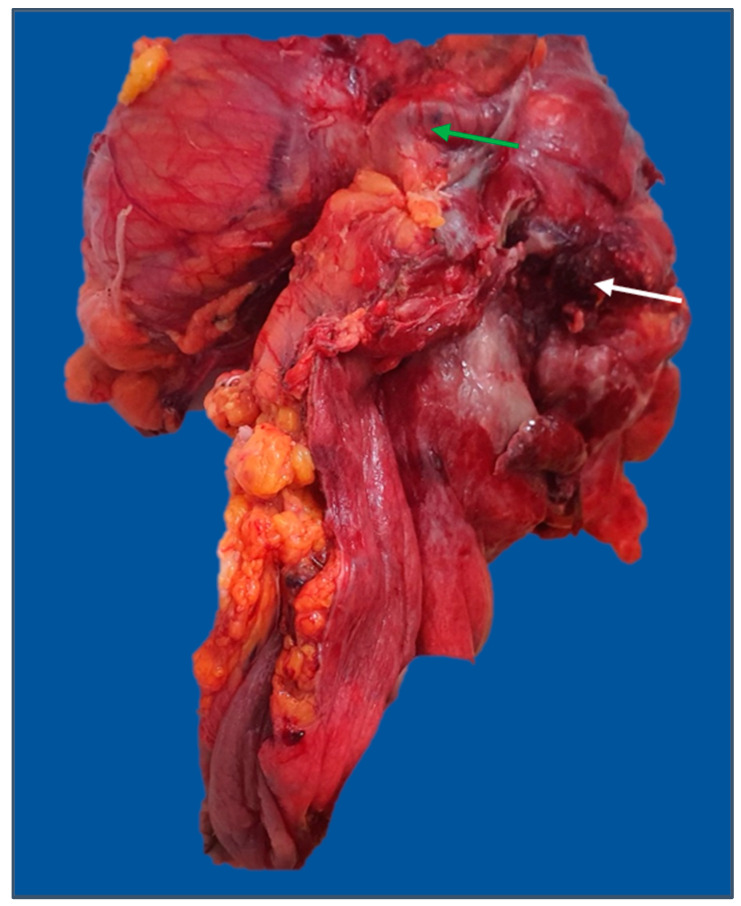
Extended left hemicolectomy specimen: en-block resection of the sigmoid colon, fallopian tube (green arrow), and left ovary for a T4 descending colon tumor with posterior perforation (white arrow). The image was used with the permission of, and was provided by, S.S.M.

The most prevalent complication was intestinal obstruction (76.1% of total cases with complications), with most of the tumors with this complication being located on the left colon (Figure 5). The main sources of intestinal hemorrhage for cases presenting with this type of complication were the rectum and rectosigmoid junction. Perforation was less frequently encountered (less than 10% of total complicated cases) (Figure 6).

Tumor locations and histopathological subtypes were found to be statistically significant in the way that the tumor locations impacted the type of complication (*p* = 0.001), and the histopathological subtypes were not uniformly distributed among the complications (*p* = 0.013). For the rest of the pathological parameters (i.e., grading, vascular and perineural invasion, and pathological stage), no statistically significant difference was found.

## 4. Discussion

Effective reduction in the incidence and mortality of colorectal cancer can be achieved through appropriate screening measures [8]. The prevalence and overall death rate of colorectal cancer have continued to decline in countries such as the United States, where screening has been implemented for several decades. According to the SEER data, age-adjusted rates for new colorectal cancer cases were falling, on average, by 1.8% each year for the period 2010–2019, with a corresponding mortality reduction of an average of 2.0% each year for the period 2011–2020 [13]. On the other hand, cancer registries worldwide, particularly in economically transitioning countries from the Eastern European Region, have been reporting significant increases in rates [14].

Despite active screening efforts, up to 33% of patients with colorectal cancer may present with symptoms requiring emergent surgical intervention. Common acute presentations include large bowel obstruction, perforation, and hemorrhage [15,16]. In our study, more than 45% of the patients had presented with a type of colorectal cancer-related complication. This high rate of patients presenting in advanced, complicated stages was attributed to deficiencies in primary and secondary prevention.

Primary prevention strategies that can further reduce risk can be informed by improving our understanding of modifiable risk factors [17]. Primary prevention efforts should focus on the promotion of physical activity, as well as encouraging healthy dietary patterns [18].

There is currently no screening program for colorectal cancer implemented at a nationwide level in our country; instead, some pilot screening programs addressing the population aged 50 to 74 years have been initiated in specific regions of the country that are characterized by high colorectal cancer mortality. The screening methods have consisted of a fecal immunochemical test (FIT) followed by a colonoscopy in cases of positive FITs [19]. Still, the adherence to these screening programs remains suboptimal, and the causes for this phenomenon are multiple. Low compliance can be explained by an inadequate level of education regarding colorectal cancer, not appreciating the benefits of screening for this malignancy, or the negative psychological effect generated by a potential positive finding.

The surgical management of these patients can be complex, requiring intraoperative decisions tailored to the situation encountered [20]. In our center, all elective surgeries were performed according to oncologic resection principles (hemicolectomies, rectal resections with total mesorectal excision, etc.), while most of the emergencies were treated according to or tailored to the type of complication. All surgical specimens were confirmed by pathological examination. The principles of oncologic resection for colorectal cancer surgery include wide radial, proximal, and distal margins and high ligation of the lymphovascular pedicle for extended lymphadenectomy (>12 nodes) [15]. Inadequate lymphadenectomy, which is referred to as “retrieved lymph nodes < 12”, was more frequent among the cases in the complications group (64.6% vs. 49.5%). This finding followed that of Elmessiry et. al, who reported that inadequate lymphadenectomy was more frequent in emergency compared to elective resection [21].

Regarding the type of surgical approach, the vast majority of the interventions were performed as open surgery (181 cases, 97.8%), whereas only a small proportion of the interventions (four cases, 2.2%) were performed as laparoscopic surgery. The predominance of open colorectal surgeries can be explained by a study in progress in our center that is evaluating the safety of anastomosis for open surgery cases only. However, several studies have supported the conclusion that both surgical approaches offer comparable long-term outcomes in terms of local recurrence and survival for patients with colorectal cancer [22,23,24].

Anastomoses were performed in both manual and stapled fashions, depending on the site of the tumor and availability. A serious postoperative complication, impacting patient outcome, is represented by anastomotic leak. Therefore, indocyanine green fluorescence angiography has emerged as a technique to prevent this complication by visualizing the bowel perfusion of the anastomotic region, as shown in a meta-analysis by Shen et. al. [25].

In our clinic, indocyanine green technology was used for the evaluation of the perfusion in the anastomotic partners starting in 2021. The KARL STORZ 4K NIR/ICG platform was used. In one case only, the use of this technique modified the level of anastomosis.

According to Yoo et al. [26], approximately 10–18% of patients with colon cancer present with obstruction at initial diagnosis [12]. Similarly, in the present study, the most frequent complication was intestinal obstruction, but the proportion of cases was significantly higher than that reported in other studies (36% of total cases). A majority of the cases were located on the left colon, followed by the rectosigmoid junction and rectum.

Different surgical options are available for resectable colon cancer with obstruction. One possibility is to perform a one-stage colectomy with regional lymph node removal. Other options include resection with diversion or diversion or stent (for selected cases), followed by colectomy [27]. A systematic review that compared colectomy to diversion followed by colectomy in left-side colonic obstructions showed that the diversion group was less likely to have a permanent colostomy. However, the study reported that there were no significant differences in the 30-day mortality and morbidity rates between the two groups [28].

The attitude adopted in our center throughout the study period for left-side colon obstructions was predominantly in the direction of two stage-resection, which consisted of treating the complication as a first step, followed by surgical resection and stoma closure for a second time.

As for the cases with perforation, they represented less than 10% of the total number of cases with complications.

Both perforation and obstruction in colon cancer tumors, along with T4 primary tumors, inadequately sampled nodes, lymphatic vessel invasion, and perineural invasion, are considered poor prognostic factors in the European Society for Medical Oncology (2020) guidelines [6].

Rectal bleeding can be a frightening experience for patients and physicians alike. Bleeding from a CRC can occur anywhere from the cecum to the distal rectum. Life-threatening hemorrhaging due to colon cancer primary is a rare occurrence, and more often, these lesions lead to chronic blood loss and anemia [20]. Our study showed a quite high incidence of inferior digestive hemorrhage, which comprised approximately 14.5% of the complicated cases, with the rectum and rectosigmoid being the main sources of bleeding.

A mention needs to be made regarding the changes in the histopathological classification of colorectal tumors since the last published edition, the 2019 World Health Organization (WHO) Classification of Tumors of the Digestive System, brought significant changes to the 2010 fourth edition, according to Ahadi et. al. Thus, this latest gastrointestinal tumor classification reflects a focus on consistency in tumor nomenclature and grading [29].

In addition to adenocarcinoma NOS, which accounts for the overwhelming majority of cases, nine other specific subtypes are recognized, all with distinct morphologies and differences in natural histories. These comprise mucinous adenocarcinoma, signet ring cell adenocarcinoma, medullary carcinoma, serrated adenocarcinoma, micropapillary adenocarcinoma, adenoma-like adenocarcinoma, adenosquamous carcinoma, carcinomas with sarcomatoid components, and undifferentiated carcinomas [29,30]. Some changes in nomenclature and the exclusion some histological variants have also been made; thus, ‘adenoma-like adenocarcinoma’, previously known as villous adenocarcinoma, and ‘carcinoma with sarcomatoid components’ are new subtypes in the WHO 2019 system. In the 2019 WHO Classification, ‘cribriform comedo-type adenocarcinoma’ and ‘spindle cell carcinoma’ are no longer accepted histological variants [29].

Similar to the data in the literature, in our study, nearly all histological variants were described among colorectal patients, with adenocarcinoma NOS accounting for the majority of cases, followed by mucinous and signet ring cell ADK. The other subtypes were less represented, accounting for less than 10 percent of the total histopathological subtypes. Interestingly, the distribution of histological subtypes among the two groups of comparison was relatively similar. This finding may largely be due to the fact that the two groups were relatively homogenous in terms of the number of patients.

In our study groups, the demographic and clinical characteristics of the patients in both groups nearly matched, but differences were observed, as expected, in the histopathological parameters, such as perineural and vascular invasion, lymph node involvement, T category, and pathological tumor stage.

As mentioned in the results section, vascular and perineural invasion were more common in the complication study group. It appeared likely that complicated tumors, being more locally advanced, showed a higher frequency for both vascular and perineural invasion.

On the same note, the presence of perineural invasion was demonstrated to be associated with a significantly worse prognosis [31]. A meta-analysis that included 58 studies and 22,900 patients also found that perineural invasion was associated with a worse 5-year OS. A follow-up of our patients over 5 or 10 years could perhaps reveal a correlation between vascular invasion and survival time, as has been shown in previous studies [9,32].

Several associated pathologies were found among the patients with colorectal cancer included in this study, including diabetes mellitus, obesity, arterial hypertension, or the presence of another neoplasm. A meta-analysis that included 54 studies and approximately nine million participants from several countries indicated a positive association between obesity and the risk of colorectal cancer development [33]. In our study, obesity was most prevalent among patients with complicated colorectal cancer. In addition, type 2 diabetes was associated with a moderately increased risk of colorectal cancer, as shown in observational studies [34], and this pathology was also prevalent among the patients in our study.

The study included the year 2020, when the COVID-19 pandemic was declared; in this regard, several studies have reported that during the initial phase of the pandemic, there was a significant decline in both the total number of surgical interventions and the number of surgeries conducted for colorectal cancer [35]. In contrast to those findings, which have shown a notable reduction in the management of cancer patients during the COVID-19 pandemic, efforts were made in our unit to maintain the same level of surgical care for colorectal cancer patients during that period of time. All patients were tested before admission and only non-emergent surgical cases were postponed until a negative PCR test was obtained.

There were several limitations to our study. First, it was a single-department retrospective study that included a relatively small number of colorectal cancer cases. The inclusion of additional centers would have increased the number of analyzed cases and, consequently, the ability to formulate more statistically significant conclusions. Secondly, no data regarding overall survival rates were collected. Nonetheless, efforts should be consistently expanded to collect more data on this topic and include other significant elements (such as molecular testing, e.g., RAS and BRAF status, microsatellite instability, and other relevant biomarkers) that contribute to an optimal diagnosis and personalized patient care.

## 5. Conclusions

Several differences were found between colorectal cancers presenting with a complication and those with no complication as complicated tumors show a more aggressive histopathologic profile and more advanced stages. It is known that a complicated tumor is associated with an unfavorable prognosis and a poorer outcome. Future studies may be able to add important data to this analysis by integrating the clinicopathological features with molecular markers and survival outcomes.

The relatively similar proportion of cases in both groups (133 patients with colorectal cancer and no complications and 117 patients with complications) indicated that there was an alarmingly high rate of patients presenting in advanced stages. Supplementary efforts must be made by healthcare systems and providers to implement a national policy for screening in both the general and susceptible populations as the cornerstone measures to reduce the rate of complicated colorectal cancers remain cancer prevention and early diagnosis.

## Figures and Tables

**Table 1:** Clinicopathological characteristics of the patients with complicated and uncomplicated CRC.

Parameter	Overall	Complicated CRC	Uncomplicated CRC	*p*-Value
No. of cases (%)	250	117 (46.8)	133 (53.2)	
Gender (%)				
● Female	97 (38.8)	50 (42.7)	47 (35.3)	0.232 ^(a)^
● Male	153 (61.2)	67 (57.3)	86 (64.7)	
Age (years, mean ± SD)	67.5 ± 9.7	67.2 ± 10.02	67.8 ± 9.4	0.623 ^(b)^
Age range	41–86	41–86	41–86	
Rural vs. urban (no. of cases, %)	122; 128 (48.8; 51.2)	62; 55 (53; 47)	60; 73 (45.1; 54.9)	0.214 ^(a)^
Tumor location (%)				0.184 ^(a)^
● Right colon	37 (14.8)	15 (12.8)	22 (16.5)	
● Transverse	14 (5.6)	6 (5.1)	8 (6)	
● Left colon	79 (31.6)	45 (38.5)	34 (25.6)	
● Rectosigmoid/rectum	120 (48)	51 (43.6)	69 (51.9)	
Type of surgery (%)				0.0003 * ^(a)^
● Radical	184 (73.6)	85 (72.6)	99 (74.4)	
● Palliative	42 (16.8)	28 (23.9)	14 (10.5)	
● Biopsy	14 (5.6)	4 (3.4)	10 (7.5)	
● None	10 (4)	0	10 (7.5)	
Tumor size (%)				0.145 ^(a)^
Mean (cm)	4.74	4.70	4.78	
● <2 cm	11 (5.8)	3 (3.2)	8 (8.2)	
● 2–5 cm	117 (61.3)	63 (67.7)	54 (55.1)	
● >5 cm	63 (33)	27 (29)	36 (36.7)	
HP type (%)				0.208 ^(a)^
● Adenocarcinoma (ADK)	202 (98.5)	95 (97.9)	107 (99.1)	
● Neuroendocrine tumor	2 (1)	2 (2.1)	0	
● Leiomyosarcoma	1 (0.5)	0	1 (0.9)	
HP subtypes (%)				0.632 ^(a)^
● Adenoma-like ADK	12 (5.9)	6 (6.3)	6 (5.6)	
● Adenosquamous carcinoma	2 (1)	1 (1.1)	1 (0.9)	
● ADK, NOS †	130 (64.4)	65 (68.4)	65 (60.7)	
● Medullary carcinoma	1 (0.5)	0	1 (0.9)	
● Micropapillary ADK	1 (0.5)	1 (1.1)	0	
● Mucinous ADK	51 (25.2)	21 (22.1)	30 (28)	
● Signet ring cell ADK	3 (1.5)	1 (1.1)	2 (1.9)	
● Serrated ADK	2 (1)	0	2 (1.9)	
Tumor grade (%)				0.252
● G1	11 (6.4)	3 (3.5)	8 (9.2)	
● G2	102 (59.3)	54 (63.5)	48 (55.2)	
● G3	59 (34.3)	28 (32.9)	31 (35.6)	
Retrieved lymph nodes (%)				0.043 * ^(a)^
● <12	99 (56.6)	53 (64.6)	46 (49.5)	
● ≥12	76 (43.4)	29 (35.4)	47 (50.5)	
Vascular invasion (%)	35 (19.6%)	23 (27.7)	12 (12.5)	0.010 * ^(a)^
Perineural invasion (%)	40 (22.3)	27 (32.5)	13 (13.5)	0.002 *
Lymph node involvement (%)	82 (46.9%)	46 (56.1)	36 (38.7)	0.021 *
pT (%)				0.002 *
● Tis	2 (1.1)	0	2 (2.1)	
● T0–T1	7 (3.8)	2 (2.4)	5 (5.1)	
● T2	30 (16.5)	6 (7.1)	24 (24.7)	
● T3	108 (59.3)	54 (63.5)	54 (55.7)	
● T4	35 (19.2)	23 (27.1)	12 (12.4)	
pN (%)				0.268
● N0	92 (50.5)	37 (43.5)	55 (56.7)	
● N1	47 (25.8)	27 (31.8)	20 (20.6)	
● N2	36 (19.8)	18 (21.2)	18 (18.6)	
● Nx	7 (3.8)	3 (3.5)	4 (4.1)	
M (%)				0.362
● M0	150 (77.7)	66 (74.2)	84 (80.8)	
● M1	39 (20.2)	20 (22.5)	19 (18.3)	
● Mx	4 (2.1)	3 (3.4)	1 (1)	
(y)pTNM (%)				0.001 *
● 0–I	30 (16.4)	4 (4.8)	26 (26.3)	
● II	55 (30.1)	28 (33.3)	27 (27.3)	
● III	59 (32.2)	32 (38.1)	27 (27.3)	
● IV	39 (21.3)	20 (23.8)	19 (19.2)	
Year (%)				0.243
● 2018	41 (16.4)	21 (17.9)	20 (15)	
● 2019	46 (18.4)	22 (18.8)	24 (18)	
● 2020	49 (19.6)	28 (23.9)	21 (15.8)	
● 2021	49 (19.6)	17 (14.5)	32 (24.1)	
● 2022	65 (26)	29 (24.8)	36 (27.1)	
Comorbidities (%)				0.006 *
● Diabetes mellitus	43 (19)	13 (12)	30 (25.4)	0.010 *
● Obesity	24 (10.6)	17 (15.7)	7 (5.9)	0.017 *
● Other neoplasm	14 (6.2)	3 (2.8)	11 (9.3)	0.041 *
● Arterial hypertension	109 (48.7)	53 (49.5)	56 (47.9)	0.803
● Other pathology	58 (28.6)	34 (35.1)	24 (22.6)	0.051

* statistically significant (*p* < 0.05); ^(a)^ Chi-square test; ^(b)^ Mann–Whitney *U*-test; ^†^ not otherwise specified.

**Table 2:** Clinical and pathological data of patients with complicated colorectal cancer.

Parameter	Obstruction	Perforation	Hemorrhage	*p*-Value
No. of cases (%)	89 (76.1%)	11 (9.4%)	17 (14.5%)	0.001 * ^(a)^
Tumor location (%)				
● Right colon	8 (9%)	5 (45.5%)	2 (11.8%)	
● Transverse	6 (6.7%)	0 (0%)	0 (0%)	
● Left colon	40 (44.9%)	3 (27.3%)	2 (11.8%)	
● Rectosigmoid/rectum	35 (39.3%)	3 (27.3%)	13 (76.5%)	
No. of cases (%)	70 (73.7%)	10 (10.5%)	15 (15.8%)	0.013 * ^(a)^
HP subtypes (%)				
● Adenoma-like ADK	4 (5.7%)	0 (0%)	2 (13.3%)	
● Adenosquamous carcinoma	0 (0%)	0 (0%)	1 (6.7%)	
● ADK, NOS †	53 (75.7%)	4 (40%)	8 (53.5%)	
● Micropapillary ADK	0 (0%)	0 (0%)	1 (6.7%)	
● Mucinous ADK	12 (17.1%)	6 (60%)	3 (20%)	
● Signet ring cell ADK	1 (1.4%)	0 (0%)	0 (0%)	
Tumor grade (%)				0.429
● G1	2 (3)	0 (0)	1 (10)	
● G2	41 (61.2)	5 (62.5)	8 (80)	
● G3	24 (35.8)	3 (37.5)	1 (10)	
Vascular invasion (%)	15 (23.1)	5 (55.6)	3 (33.3)	0.115
Perineural invasion (%)	23 (35.4)	4 (44.4)	0	0.075
Lymph node involvement (%)	35 (54.7)	8 (80)	3 (37.5)	0.174
No. of cases (%)	68 (81)	6 (7.1)	10 (11.9)	0.211
(y)pTNM (%)				
● 0–I	2 (2.9)	0	2 (20)	
● II	24 (35.3)	1 (16.7)	3 (30)	
● III	27 (39.7)	2 (33.3)	3 (30)	
● IV	15 (22.1)	3 (50)	2 (20)	
No. of cases (%)	89 (76.1)	11 (9.4)	17 (14.5)	0.446
Year (%)				
● 2018	18 (20.2)	0	3 (17.6)	
● 2019	17 (19.1)	3 (27.3)	2 (11.8)	
● 2020	23 (25.8)	3 (27.3)	2 (11.8)	
● 2021	13 (14.6)	1 (9.1)	3 (17.6)	
● 2022	18 (20.2)	4 (36.4)	7 (41.2)	

* statistically significant (*p* < 0.05); ^(a)^ Chi-square test; ^†^ not otherwise specified.

## Data Availability

The data presented in this study are available on request from the corresponding author.
